# Supplementary material for: ProteinShader: illustrative rendering of macromolecules
Source: BMC Struct Biol. 2009 Mar 30;9:19. doi: 10.1186/1472-6807-9-19 (PMC2672931; doi:10.1186/1472-6807-9-19)
Supplement: Additional file 1 — ProteinShader program without source code. This compressed file contains the complete ProteinShader program including associated libraries, but no source code. A README.txt file gives an overview of the ProteinShader distribution, and the index.html file in the help subdirectory has directions on getting started with the program as well as a set of tutorials. [file 1472-6807-9-19-S1.zip › ProteinShader-beta-0_9_4-binary/help/api/org/proteinshader/graphics/Cylinder.html]

Cylinder (ProteinShader API)


|  |  |  |
| --- | --- | --- |
| **Overview** \| **Package** \| **Class** \| **Use** \| **Tree** \| **Deprecated** \| **Index** \| **Help** | |  |
| PREV CLASS   **NEXT CLASS** | **FRAMES**    **NO FRAMES**     **All Classes** |  |
| SUMMARY: NESTED \| FIELD \| CONSTR \| METHOD | DETAIL: FIELD \| CONSTR \| METHOD |  |


---


## org.proteinshader.graphics Class Cylinder

```
java.lang.Object
  org.proteinshader.graphics.Shape
      org.proteinshader.graphics.Cylinder
```

---

``` public class Cylinder extends Shape ```

Knows how to draw a simple cylinder useful for representing the bonds
between atoms. The cylinder can be capped with a small sphere at one
or both ends. To speed up rendering, methods are provided for
creating and using OpenGL display lists.

---

| **Field Summary** | |
| --- | --- |
| `static int` | `MIN_CAP_TILING`             The minumum tiling (number of slices and stacks) for a capping sphere is 3. |
| `static int` | `MIN_SLICES`             The minimum number of slices for a cylinder is 3. |
| `static int` | `MIN_STACKS`             The minimum number of stacks for a cylinder is 1. |

**Fields inherited from class org.proteinshader.graphics.Shape**

`HALF_PI, PI, SPECULAR_COLOR, TWO_PI`


**Constructor Summary**

`Cylinder()`
  
          Constructs a Cylinder.


| **Method Summary** | |
| --- | --- |
| `CylinderListInfo` | `createDisplayList(GL gl, CylinderListInfo info)`             Creates an OpenGL display list for drawing a cylinder with the radii, slices, stacks, and end caps specified in the CylinderListInfo object given as an argument. |
| `int` | `createDisplayList(GL gl, double baseRadius, double topRadius, double height, int slices, int stacks, boolean capBase, boolean capTop, int capSlices, int capStacks)`             Stores the OpenGL commands for drawing a cylinder as an OpenGL display list. |
| `void` | `draw(GL gl, double baseRadius, double topRadius, double height, int slices, int stacks, boolean capBase, boolean capTop, int capSlices, int capStacks)`             Draws a cylinder oriented along the z-axis, with the base of the cylinder at the origin and the top of the cylinder at xyz = (0, 0, height). |

**Methods inherited from class org.proteinshader.graphics.Shape**

`deleteDisplayList, executeDisplayList, executeDisplayList, setMaterial`

**Methods inherited from class java.lang.Object**

`clone, equals, finalize, getClass, hashCode, notify, notifyAll, toString, wait, wait, wait`

**Field Detail**

### MIN\_SLICES

```
public static final int MIN_SLICES
```

:   The minimum number of slices for a cylinder is 3.

    **See Also:**: Constant Field Values

---


### MIN\_STACKS

```
public static final int MIN_STACKS
```

:   The minimum number of stacks for a cylinder is 1.

    **See Also:**: Constant Field Values

---


### MIN\_CAP\_TILING

```
public static final int MIN_CAP_TILING
```

:   The minumum tiling (number of slices and stacks) for
    a capping sphere is 3.

    **See Also:**: Constant Field Values


**Constructor Detail**

### Cylinder

```
public Cylinder()
```

:   Constructs a Cylinder.
    A GLU quadrics object to be used for drawing cylinders is created
    here. The quadrics object is set to use GLU\_FILL for a drawing
    style, GLU\_OUTSIDE for orientation, and GLU\_SMOOTH for the surface
    normals.


**Method Detail**

### createDisplayList

```
public CylinderListInfo createDisplayList(GL gl,
                                          CylinderListInfo info)
```

:   Creates an OpenGL display list for drawing a cylinder with
    the radii, slices, stacks, and end caps specified in the
    CylinderListInfo object given as an argument.

---


### createDisplayList

```
public int createDisplayList(GL gl,
                             double baseRadius,
                             double topRadius,
                             double height,
                             int slices,
                             int stacks,
                             boolean capBase,
                             boolean capTop,
                             int capSlices,
                             int capStacks)
```

:   Stores the OpenGL commands for drawing a cylinder as an OpenGL
    display list.

    :   **Parameters:**: `gl` - the current GL object.: `baseRadius` - the radius of the base of the cylinder.: `topRadius` - the radius at the top of the cylinder.: `height` - the height of the cylinder.: `slices` - number of slices in the cylinder (at least 3).: `stacks` - number of stacks in the cylinder (at least 1).: `capBase` - determines if base is capped by a sphere.: `capTop` - determines if top is capped by a sphere.: `capSlices` - number of slices for cap sphere (at least 3).: `capStacks` - number of stacks for cap sphere (at least 3). **Returns:**: The name (an integer) of the OpenGL display list.

---


### draw

```
public void draw(GL gl,
                 double baseRadius,
                 double topRadius,
                 double height,
                 int slices,
                 int stacks,
                 boolean capBase,
                 boolean capTop,
                 int capSlices,
                 int capStacks)
```

:   Draws a cylinder oriented along the z-axis, with the base of
    the cylinder at the origin and the top of the cylinder at
    xyz = (0, 0, height). The cylinder is open at both the base
    and the top, unless a cap (a small sphere) is requested at
    one or both ends.

    :   **Parameters:**: `gl` - the current GL object.: `baseRadius` - radius of the base of the cylinder (usually 1).: `topRadius` - radius at the top of the cylinder (usually 1).: `height` - the height of the cylinder.: `slices` - the number of slices in the cylinder.: `stacks` - the number of stacks in the cylinder.: `capBase` - determines if base is capped by a sphere.: `capTop` - determines if top is capped by a sphere.: `capSlices` - number of slices for cap sphere (no less than 3).: `capStacks` - number of stacks for cap sphere (no less than 3).


---


|  |  |  |
| --- | --- | --- |
| **Overview** \| **Package** \| **Class** \| **Use** \| **Tree** \| **Deprecated** \| **Index** \| **Help** | |  |
| PREV CLASS   **NEXT CLASS** | **FRAMES**    **NO FRAMES**     **All Classes** |  |
| SUMMARY: NESTED \| FIELD \| CONSTR \| METHOD | DETAIL: FIELD \| CONSTR \| METHOD |  |


---

# *Copyright © 2007-2008*
